# High expression of QSOX1 reduces tumorogenesis, and is associated with a better outcome for breast cancer patients

**DOI:** 10.1186/bcr3341

**Published:** 2012-10-25

**Authors:** Nicolas Pernodet, François Hermetet, Pascale Adami, Anne Vejux, Françoise Descotes, Christophe Borg, Marjorie Adams, Jean-René Pallandre, Gabriel Viennet, Frédéric Esnard, Michèle Jouvenot, Gilles Despouy

**Affiliations:** 1 Université de Franche-Comté, EA3922 « Estrogènes, Expression Génique et Pathologies du Système Nerveux Central », IFR133, U.F.R. Sciences et Techniques, 16 route de Gray, 25030 Besançon Cedex, France; 2 Université de Bourgogne, EA7270, Equipe 'Biochimie du Peroxysome, Inflammation et Métabolisme Lipidique' (Bio-peroxIL), 6 Bd Gabriel, 21000 Dijon, France; 3 Service de Biochimie Biologie Moléculaire Sud, Centre Hospitalier Lyon sud, Hospices Civils de Lyon, Pierre Bénite, France; 4 Université de Franche Comté, UMR INSERM-UFC-EFS 1098 « Relation Hôte Greffon et Ingénierie Cellulaire et Génique », 240 route de Dôle, 25000 Besançon, France; 5 Université François Rabelais, INSERM U1100, 10 boulevard Tonnellé, 37000 Tours, France

## Abstract

**Introduction:**

The gene quiescin/sulfhydryl oxidase 1, *QSOX1*, encodes an enzyme directed to the secretory pathway and excreted into the extracellular space. QSOX1 participates in the folding and stability of proteins and thus could regulate the biological activity of its substrates in the secretory pathway and/or outside the cell. The involvement of QSOX1 in oncogenesis has been studied primarily in terms of its differential expression in systemic studies. QSOX1 is overexpressed in prostate cancers and in pancreatic adenocarcinoma. In contrast, *QSOX1 *gene expression is repressed in endothelial tumors. In the present study, we investigated the role of *QSOX1 *in breast cancer.

**Methods:**

We analyzed QSOX1 mRNA expression in a cohort of 217 invasive ductal carcinomas of the breast. Moreover, we investigated QSOX1's potential role in regulating tumor growth and metastasis using cellular models in which we overexpressed or extinguished QSOX1 and xenograft experiments.

**Results:**

We showed that the *QSOX1 *expression level is inversely correlated to the aggressiveness of breast tumors. Our results show that QSOX1 leads to a decrease in cell proliferation, clonogenic capacities and promotes adhesion to the extracellular matrix. QSOX1 also reduces the invasive potential of cells by reducing cell migration and decreases the activity of the matrix metalloproteinase, MMP-2, involved in these mechanisms. Moreover, *in vivo *experiments show that QSOX1 drastically reduces the tumor development.

**Conclusions:**

Together, these results suggest that QSOX1 could be posited as a new biomarker of good prognosis in breast cancer and demonstrate that QSOX1 inhibits human breast cancer tumorogenesis.

## Introduction

The Quiescin Sulfhydryl Oxidase 1 (*QSOX1*) gene was identified by our group in primary culture of guinea pig endometrial glandular epithelial cells [1]. The human gene is located on chromosome 1 (1q24) and encodes two major isoforms by alternative RNA splicing: QSOX1S (66 kDa) and QSOX1L (82 kDa) [2]. The short transcript appears to be ubiquitous, whereas the expression of the longer form seems to be tissue specific [3]. The longer form of the QSOX1 protein retains a potential transmembrane segment that could allow for anchorage to the membrane. The QSOX1 N-terminus contains a sequence targeting the nascent protein to the endoplasmic reticulum. Moreover, no signal for permanent retention in the endoplasmic reticulum (KDEL sequence) was identified, suggesting an extracellular destination [4]. In addition, QSOX1 proteins have been detected in the endoplasmic reticulum, the Golgi apparatus and the secretion vesicles [5]. These proteins can also be found in culture supernatant and in extracellular spaces, confirming that they are secreted [1].

QSOX1 is the product of an ancient fusion between thioredoxin domains and Flavin Adenine Dinucleotide (FAD) -binding module, ERV/ALR. A first CXXC motif is located in N-terminus and can act as a reducer or an oxidant. The other CXXC motif is located in a FAD domain within C-terminus [6]. The QSOX1 protein belongs to a family of FAD sulfhydryl oxidases and catalyzes the oxidation of thiols to disulfides. *In vitro*, enzymatic studies on avian QSOX1 have demonstrated that this enzyme is able to both catalyze disulfide bridges of a large array of monothiol substrates (such as glutathione) and reduce proteins and peptides [7,8]. Moreover, it seems that QSOX1 is not a disulfide isomerase but instead assists the Protein Disulfide Isomerase (PDI) by establishing disulfide links in mature proteins [9,10].

Previous reports showed that serum depletion-induced quiescence, as well as cell contact inhibition, led to a QSOX1 mRNA accumulation in guinea pig endometrial glandular epithelial cells [1] and in human lung fibroblasts [3]. These experimental data suggest that QSOX1 could be involved in the negative control of the cell cycle. Furthermore, in our laboratory it was demonstrated that over-expression of guinea pig QSOX1-S in MCF-7 cells decreased the cellular proliferation and protected cells against oxidative stress [11]. It is now known that cellular damage due to an accumulation of Reactive Oxygen Species (ROS) leads to tumorogenesis [12,13]. By the reducing activity of its first CXXC motif, QSOX1 could prevent tumorogenesis by down-regulating ROS levels in cells.

Another study suggested that QSOX1 could take part in the cell anchorage mechanism. Indeed, increased mRNA levels have been detected in human lung fibroblast when cell/plate or cell/cell adhesion was disturbed by a mechanical or chemical action [14].

Several systemic studies have demonstrated an alteration of *QSOX1 *expression in cancer cell models. In fact, one study demonstrated the presence of peptide fragments of QSOX1 at highly significant rates in plasma from patients suffering from pancreatic cancer [15]. Moreover, very recently, it was reported that QSOX1 could promote invasion of pancreatic tumor cell lines by activating matrix metalloproteinase [16]. In another, a correlation was observed between the overexpression of *QSOX1 *and the initiation of prostate tumor growth [17]. On the other hand, *QSOX1 *expression is repressed by epigenetic regulation, especially by histone deacetylation in a cell model of endothelial tumors. Moreover, this down-regulation seems to be necessary for angiogenesis, an essential phenomenon for metastasis development [18]. These data suggest an involvement of QSOX1 in the mechanisms of carcinogenesis.

In the present study, QSOX1 mRNA expression was investigated in a retrospective cohort of 217 invasive ductal carcinomas (IDC) of the breast. The impact of the QSOX1 expression on characteristic phenotypes of breast cancer cells and tumor growth was subsequently determined.

## Materials and methods

### Clinical analysis

#### Patients and tumor characteristics (Table 1)

The study included a retrospective cohort of 217 patients with invasive ductal carcinomas of the breast. This cohort was derived from the population described previously [19]. Estrogen receptor (ER) and progesterone receptor (PgR) were assayed in cytosol using the radioligand reference method [20]. PAI-1 (Plasminogen activator inhibitor-1) was measured in Triton extract by the enzyme-linked immunoassay (PAI-1, Imubind #821, American Diagnostica, Stamford, CT, USA) as elsewhere described [21]. Studies involving human primary breast tumors were performed according to the principles embodied in the Declaration of Helsinki. Tissue biopsies were obtained as part of surgical treatments for the hormone receptor content determination. Remaining samples were included anonymously in this study. Ethical approval and consent were not required due to the routine nature of the procedure.

#### Reverse Transcriptase Quantitative PCR (RT-qPCR) analysis

Detailed information on RNA extraction and RTqPCR has been previously described [19]. The QSOX1 primers used were: forward, 5'-GGAAGCTT CTGGAAGTCGTG-3' and reverse, 5'-CAAAAGACCAGGCTCAGAGG-3' for amplification of a 211 bp fragment (GenBank NM_002826). The QSOX1 target concentration was expressed relative to the concentration of the GAPDH housekeeping gene [19].

#### Statistical analysis

The median follow-up at the time of analysis was 54 months (range: 2 to 109). Patients were followed up for metastasis relapse (nodal or distant metastasis and local recurrence were relapse). Analysis of the distribution of QSOX1 RNA expression in relation to usual prognostic parameters was performed using the Mann-Whitney or Kruskall Wallis test. Metastasis free survival probabilities were estimated using Kaplan Meier estimators and were compared using the log-rank test. These analyses were performed with the SPSS software version 17.0 (IBM, Armonk, NY, USA).

### Experimental analysis

#### Reagents and antibodies

Cell culture reagents were purchased from Invitrogen (Cergy Pontoise, France). Miscellaneous reagents were purchased from Sigma Aldrich (L'Isle d'Abeau Chesnes, France). Specific inhibitor of matrix metalloproteinase (MMP)2 was purchased from Millipore (Molsheim, France). The following antibodies were used: for Western blotting, polyclonal anti-rat QSOX1 [4] diluted at 1:7,500, for immunohistochemistry, polyclonal anti-human QSOX1 (Proteintech Group, Inc., Chicago, IL, USA) diluted at 1:100, polyclonal anti-MMP-2 (Cell Signaling Technology, Danvers, MA, USA) diluted at 1:2,000, polyclonal anti-actin (Sigma Aldrich) diluted at 1:5,000 and polyclonal anti-rabbit (P.A.R.I.S, Compiègne, France) diluted at 1:10,000.

#### Cell culture

Cells were cultured in DMEM (Dulbecco's Minimum Essential Medium) supplemented with 2 mM L-Glutamine, 100 μg/ml penicillin, 100 μg/ml streptomycin and 5% fetal calf serum (FCS) and kept in a humidified 5% CO_2_water saturated atmosphere. Cell viability was estimated by counting Trypan blue excluding cells.

#### Plasmid construction, small-hairpinRNA (shRNA) experiments and transfection

The pcDNA3.1-QSOX1S plasmid was constructed by cloning the coding sequence of QSOX1-S splice variant 2 (AF361868) between the EcoRV and BamHI sites of pcDNA™3.1/Hygro^(-) ^(Invitrogen). MCF-7 cells were transfected with the pcDNA3.1 and the pcDNA3.1-QSOX1S expression plasmids using Lipofectamine 2000 (Invitrogen), according to the manufacturer's instructions. Then, cells were selected with 200 μg/ml Hygromycin B (PAA, Les Mureaux, France).

shRNA vectors from the Mission human shRNA hQSOX1 clone sets (Sigma Aldrich) were used: shQSOX1-1 (TRCN0000064183), shQSOX1-2 (TRCN0000064185), and appropriate control vector: shC (SHGLYNM_001004128). Lipofectamine LTX (Invitrogen) was used to stably transfect MDA-MB 231 cells, according to the manufacturer's recommendations. Clones were selected with 1 μg/ml Puromycin (Sigma Aldrich).

#### QSOX1 mRNA detection and level analysis

Total RNAs were extracted as previously described [11]. RTqPCR was performed with the Step One Real Time PCR System (Applied Biosystems, Carlsbard, CA, USA), using the SYBER Green PCR Master Mix (Applied Biosystems). Target (endogenous and plasmid encoded QSOX1 mRNA) and endogenous control (H3.3 like histone H3B-2 (*H3B-2)*) amplifications exhibited 100 ± 5% efficiency (R^2 ^>99% for the standard curve); *QSOX1 *primer sequences were: hQSOXE+Ts1: 5'-GCCACCCTCAACTTCCTCAAG-3', hQSOXE+Trev1: 5'-ACCCAGCTGCAGGGAAGTC-3'; *H3B-2 *primer sequences were: HisI: 5'-GCTAGCTGGATGTCTTTTGG-3', HisN: 5'-GTGGTAAAGCACCCAGGAA-3'). Each sample was analyzed in triplicate and then differences in the expression of each gene were quantified using the ΔΔCt approach using endogenous control.

#### Immunoblotting

SDS-polyacrylamide gel electrophoresis and transfer of proteins onto PVDF membranes (Bio-Rad, Marnes-la-Coquette, France) were performed using standard protocols. The antibodies were used at the previously indicated dilution. Immunoreactive bands were detected using goat horseradish peroxidase (HRP)-coupled secondary anti-rabbit antibodies (1:10,000 in antibody blocking buffer) and ECL Plus reagent (GE Healthcare Life Sciences, Saclay, France), according to the manufacturer's protocol.

Blots were stripped by incubation in stripping buffer (62.5 mM Tris, pH 6.7, 100 mM β-mercaptoethanol and 2% SDS) for 30 minutes at 50°C, blocked again in TBST buffer containing 5% non-fat milk and then probed a second time with polyclonal anti-actin.

#### Immunohistochemistry (IHC)

Formalin-fixed, paraffin-embedded tissue blocks from patients whom underwent surgical resection for invasive ductal carcinoma and normal breast tissue were sectioned at 5 μm thickness using water flotation for tissue section transfer and dried overnight at room temperature. The slides were de-waxed, rehydrated and subjected to heat induced epitope retrieval using a proprietary citrate based retrieval solution for 40 minutes. Endogenous peroxidases were blocked. QSOX1 detection was performed as described in Antwi *et al. *[15]. QSOX1 location analysis was performed by Dr. Viennet, a board-certified pathologist.

#### Cell-Matrix Adhesion Assay

Cells were incubated at 37°C for 60 minutes at a density of 4 × 10^5 ^cells/well in serum-free DMEM on non-coated well plates (three wells/cell line: one well to control the number of seeded cells and the two others for technical duplicates). After washing, adherent cells were collected and pelleted down. Then, cells were counted with Malassez cell. Results were expressed as the ratio between plate adhering cells and total seeded cells and reported as mean ± SD of triplicate determinations.

#### Colony formation assay

Cells were plated in six-well tissue culture plates at a density of 50 cells/cm². After 28 days, colonies were fixed with ethanol and stained with 2% crystal violet, washed with water to remove the excess dye, and imaged by a scanner. Quantitative changes in clonogenicity were determined by counting the colonies, using Bio-Rad *Vision-Capt *software.

#### Anchorage independent cell proliferation

Cells were seeded at a density of 6 × 10^4 ^cells per 35-mm cell culture dish in 0.3% agar. After 28 days, the top agar cell layers were covered with culture medium containing 5% FCS. Images from four representative fields of each well were taken and analyzed.

#### Cell migration and invasion assay

For the migration assay, 10^5 ^cells were suspended in 300 μL serum-free medium and added into the upper chamber of the Boyden modified chamber™ (SPL Life Sciences, Pocheon-si, Korea). For the invasion assay, 10^5 ^cells in 200 μL serum-free medium, in the presence or absence of 10 μM of a MMP-2 specific inhibitor, *cis-*9-Octadecenoyl-N-hydroxylamide (OA-Hy) [22,23], were added into the upper chamber. Five hours before cell seeding, 50 μL of extra cellular matrix (ECM) gel (1 mg/ml) were added to the upper chamber. Thereafter, the cells were incubated 24 h for migration or invasion assay. The cells on the upper surface were removed using a cotton bud. The remaining invading cells were fixed, stained with 2% crystal violet and the images from four representative fields of view (FOV) of each membrane were taken. The invasive cells in the lower chamber were counted.

#### Zymography assay

The gelatinase activity of MMPs in the serum-free media was analyzed by gelatin-zymography. A total of 10^5 ^cells suspended in 250 μl were plated onto a 24-well plate. The serum-free medium was collected after 12 h of incubation for MCF-7 cells and 24 h for MDA-MB-231 cells. HT-1080 conditioned media was used as positive control. The proteins from the conditioned media were 100-fold concentrated by acetone precipitation and solubilized in Laemmli Buffer (without β-mercaptoethanol). Then, proteins were separated by SDS PAGE electrophoresis using 10% (w/v) acrylamide gels containing 1% (w/v) gelatin at 20 mA. Gels were rinsed in Triton X-100 (2.5%, v/w) and incubated in 50 mM Tris-HCl, 150 mM CaCl_2 _and 5 μM ZnCl_2 _(pH 8.0) at 37°C for 16 h. Gels were stained with 0.25% (w/v) Coomassie brilliant blue. Digested areas appeared clear on a blue background, indicating the location of active MMPs.

### Xenograft experiments

CIEA NOG mice were obtained from Taconic (Germantown, NY, USA) and maintained in the UMR1098 animal facility (agreement number #C25-056-7). Approval for animal experimentation and care was received from the Services Vétérinaires de la Santé et de la Protection Animale delivered by the Ministère de l'Agriculture, Paris, France and experimental procedures were approved by a local ethic committee.

A total of 2 × 10^6 ^cells of different cell lines (MDA-MB-231 shC and MDA-MB-231 shQSOX1-1) resuspended in 100 μL of PBS per mouse were inoculated subcutaneously in NOG mice (*n *= 5 per group) and tumor growth was monitored biweekly in each group. Tumor volume was calculated by the formula *V = *½ *a × b^2^*, where *a *is the longest tumor axis and *b *is the shortest tumor axis. When tumors reached 1 cm in diameter, mice were sacrified and each tumor was fixed in formol and photographed. During the sacrifice, photos have been taken in order to keep proof of where tumors developed.

## Results

### QSOX1 localization in normal and in breast cancer tissues

**Figure 1:**
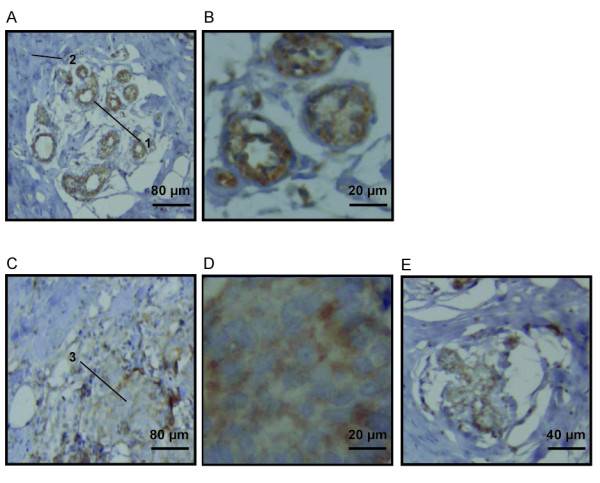
**QSOX1 localization in normal and breast cancer tissues**. Anti-QSOX1 IHC on normal mammary gland section (**A, B**) and on invasive ductal carcinoma (IDC) section (**C, D, E**). (A) The first arrow (1) indicates the presence of labelling in normal breast ductulo-lobular units. (B) Perinuclear and apical staining in ductulo-lobular unit cells. (C) The second arrow (2) indicates a labelling in tumor cells from IDC and the third arrow (3) shows the absence of labelling in stroma cells. (D) Diffuse cytoplasmic staining in tumors cells. (E) Perinuclear and apical labelling in non cancerous cells from IDC.

IHC experiments were performed on sections of normal mammary gland and invasive ductal carcinoma (IDC). Results in Figure 1 show a QSOX1 expression in human normal breast tissue: QSOX1 is localized at the level of milk ducts and channels of the ductulo-lobular units (Figure 1A); the labelling is perinuclear and at the apex of the constituent cells of these histological structures (Figure 1B), suggesting a localization in different compartments of the secretory pathway. In the tissues from IDC, a diffuse cytoplasmic labelling of QSOX1 was observed in tumor cells (Figure 1C, D). In non-cancerous cells from IDC tissues section, QSOX1 labelling appeared as a punctate perinuclear and apical staining in the ductulo-lobular units (Figure 1E), as observed in normal mammary glands. A lack of labelling was noted in stromal cells and adipocytes, whether observing sections from normal or pathological tissue (Figure 1A, C).

### QSOX1 mRNA expression in breast cancer

**Figure 2:**
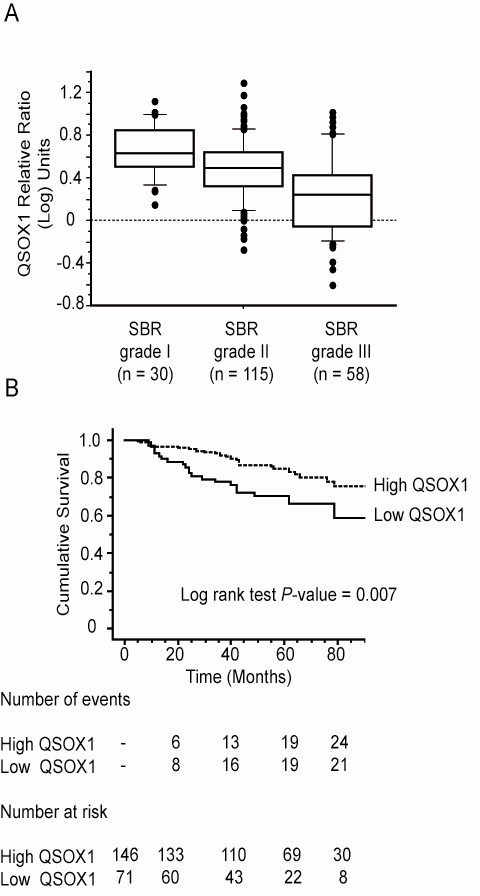
**Expression of QSOX1 mRNA in a cohort of 217 invasive ductal carcinomas of the breast**. (**A**) Box plots of QSOX1 expression in relation to the histological grade (Kruskall Wallis test, *P *<0.001); (**B**) Kaplan-Meier curves for metastasis-free survival probabilities according to QSOX1 expression categorized with a cut-off equal to the upper threshold of the first tertile. Low: < first tertile, High: ≥ second tertile. SBR: Scraff Bloom Richardson;

We investigated QSOX1 mRNA expression in a cohort of 217 invasive ductal carcinomas of the breast. The mean QSOX1 value measured by RTqPCR was 3.56 and the median was 2.84 (range: 0.25 to 19.89). Table 1 shows the median value of QSOX1 in relation to tumor characteristics that are usually linked to the prognosis. Indeed, the median QSOX1 expression value was significantly lower in the patients with pejorative prognostic factors: premenopausal status, SBR grade III, negative ER or PgR, and/or high PAI1 level (Figure 2A, Kruskall Wallis test, *P*-value <0.001).

**Table 1:** QSOX1 mRNA expression in relation to the usual prognostic factors in 217 breast IDC

Characteristics		n	%	median	*P*-value
**Menopausal status**	Pre	71	32.7	2.56	
	Post	146	67.3	3.31	0.003

**Tumor size (mm)**	<20	90	41.5	3.08	
	≥20	120	55.3	2.62	0.229
	ND	7	3.2	3.38	

**Lymph node status **	pN0	101	46.5	3.18	
	pN+	116	53.5	2.64	0.444

**SBR Grade**	I	30	13.8	4.25	
	II	115	53.0	3.14	
	III	58	26.7	1.74	<0.001
	ND	14	6.5	2.42	

**ER status**	Positive	179	82.5	3.17	
	Negative	38	17.5	1.51	<0.001

**PgR status**	Positive	169	77.9	3.17	
	Negative	48	22.1	1.97	<0.001

PAI-1 status*	Low	153	70.5	3.18	
	High	44	20.3	1.46	<0.001
	ND	20	9.2	2.77	

*P*-values correspond to Mann-Whitney test or Kruskall Wallis test. ER, estrogen receptor; ND, Not Determined; PgR, Progesteron receptor; SBR, Scarff Bloom Richardson. *Low: ≤75% quartile; High: >75% quartile.

The Kaplan Meier curves were constructed after segmentation into two groups on the basis of the QSOX1 expression cut-off equal to the upper threshold of the first tertile. This cut-off, that is, 2.11, allowed the discrimination between high and low QSOX1 status. It was observed that high values of QSOX1 expression were related to a good prognosis (Figure 2B, P-value = 0.007).

### Expression of hQSOX1 in breast cancer cell lines

**Figure 3:**
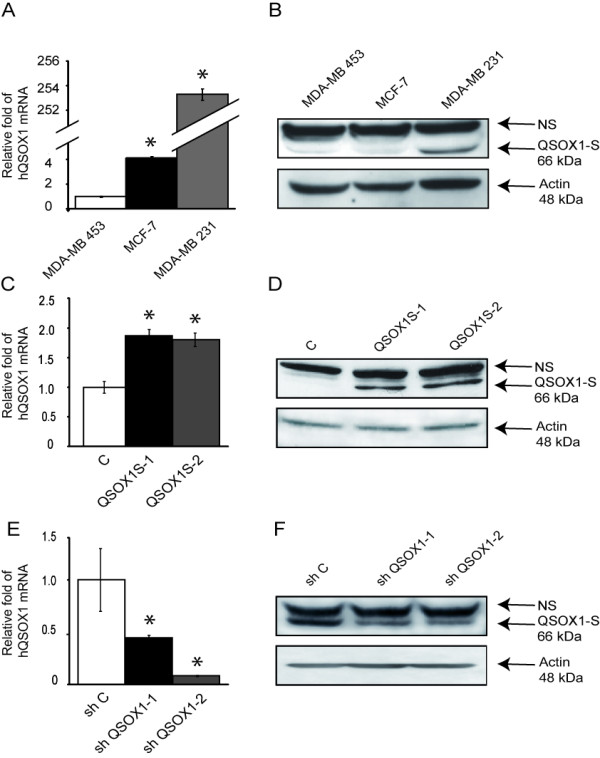
**Analysis of QSOX1 expression in breast cancer cell lines**. (**A, C, E**) After reverse transcription, relative QSOX1 mRNA expressions were determined by qPCR (C: MCF-7; E: MDA-MB-231). H3B-2 mRNA was used for normalization. Data are means ± S.D. of three independent experiments. *P <0.05, compared to control (Wilcoxon test). (**B, D, F**) Western blot analysis of QSOX1 expression. Total proteins (50 μg) were separated on SDS-PAGE and transferred to PVDF membranes (D: MCF-7; F: MDA-MB-231). Proteins were detected using anti-QSOX1 and anti-actin antibodies. NS, Non Specific.

Coppock and coworkers have previously observed, by Northern blotting, that QSOX1 is expressed at a low level in MCF-7 and MDA-MB-453 cells and at a high rate in MDA-MB-231 breast cancer cell lines [3]. First, we analyzed expression of the *QSOX1 *gene by RTqPCR in these cell lines. Our results indicate that the relative fold of QSOX1 expression in MDA-MB-231 and MCF-7 cells was 253.5 ± 1 (mean ± SD) and 4.2 ± 0.5, respectively compared to MDA-MB-453 cells (Figure 3A). These results were confirmed at the protein level. In fact, QSOX1S expression is very weak in MDA-MB-453 and MCF-7 cells and high in MDA-MB-231 cell lines (Figure 3B).

To investigate the function of hQSOX1S in the breast cancer cell line, we established stably transfected monoclonal MCF-7 cells overexpressing hQSOX1S (MCF-7 QSOX1S-1 and MCF-7 QSOX1S-2 lines). We then measured the level of QSOX1S expression in MCF-7 Control (MCF-7 C) and MCF-7 QSOX1S-1 and MCF-7 QSOX1S-2 lines under standard culture conditions. The relative fold of QSOX1 expression was determined by RTqPCR. MCF-7 QSOX1S-1 and MCF-7 QSOX1S-2 lines showed an increase of 186% ± 11 (mean ± SD) and 180% ± 11, respectively, compared to MCF-7 C cells (Figure 3C). QSOX1 overexpression was also assessed at the protein level. The signal corresponding to hQSOX1S was strongly increased in MCF-7 QSOX1S-1 and MCF-7 QSOX1S-2 lines as compared to MCF7 C (Figure 3D).

Our second approach to studying the function of QSOX1 consisted of silencing QSOX1 expression. A series of RNA interference experiments using shRNA vectors were then performed in MDA-MB-231 cells. After RTqPCR analysis, two shRNA QSOX1 vectors (shQSOX1-1 and shQSOX1-2), inducing 55% ± 4 (mean ± SD) and 92% ± 1 decreases of the QSOX1 expression, respectively, were selected (Figure 3E). Results obtained by Western blotting showed a decrease in the signal corresponding to QSOX1S in the knock-down cell lines compared to the control (Figure 3F). MDA-MB-231 shQSOX1-2 harbors a higher extinction of the protein than MDA-MB-231 shQSOX1-1, as observed at the mRNA level.

### QSOX1 decreased cell proliferation and colony formation

**Figure 4:**
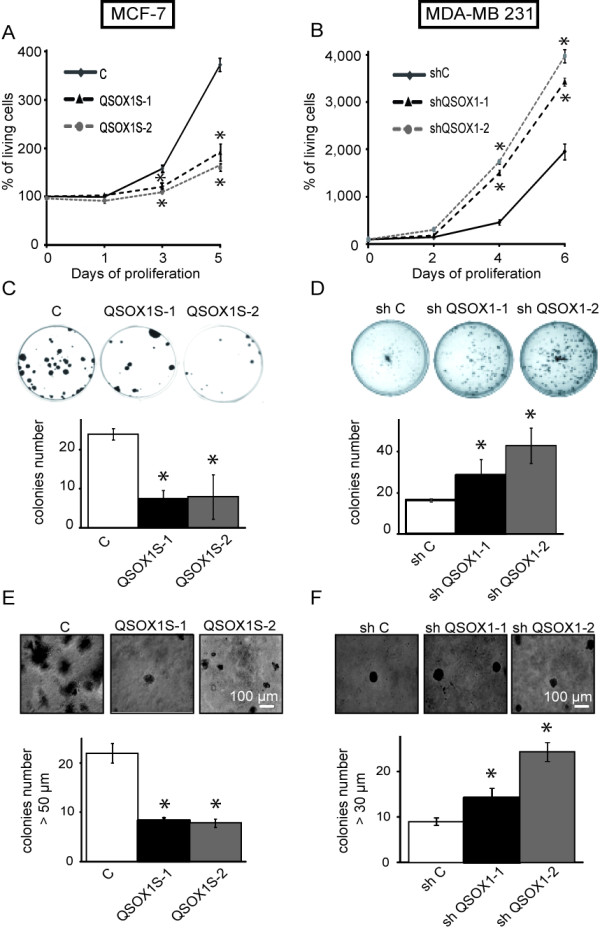
**Effect of QSOX1 on cell proliferation and colony formation**. (**A, B**) Cells were plated at 5,000 cells/cm². Cell viability was estimated by counting Trypan blue excluding cells. Data are expressed relative to the living cell number at day 0. Values are expressed as mean ± SD for three experiments. (**C, D**) For a colony formation assay, 500 cells were plated and 28 days later, cells were fixed and stained with 2% crystal violet. Quantitative changes were evaluated by counting using *Vision Capt *software. (**E, F**) Colony assays in soft agar were done to evaluate the effects of QSOX1 on anchorage independent cell proliferation. Cells were embedded in 0.3% agar and the agar cell layers were covered with 1 ml medium containing 5% FCS. After 28 days of growth, the colonies were photographed. Pictures of colonies were superposed with a Malassez cell picture in order to determine colony size. Results indicated number and colony size from four representative fields of each well. * *P *<0.05, compared to control (Wilcoxon test). These experiments are representative of three separate assays.

Cancer is characterized by an enhancement of cell proliferation. To determine the involvement of QSOX1 in cell proliferation, 10^4 ^cells were initially seeded and then the live cell number was measured. MCF-7 QSOX1S-1 and MCF-7 QSOX1S-2 proliferated less than MCF-7 C. Indeed, after five days of culture, living cell number increased 3.6-fold for MCF-7 C, 1.9 for MCF-7 QSOX1S-1 and 1.8 for MCF-7 QSOX1S-2 (Figure 4A). After one week of culture, the number of MDA-MB-231 shQSOX1-1 or shQSOX1-2 living cells was strongly increased compared to MDA-MB-231 shC (Figure 4B). In fact, the living cell number increased 19.6-fold for MDA-MB-231 shC, 34.3-fold for MDA-MB-231 shQSOX1-1 and 39.8-fold for MDA-MB-231 shQSOX1-2.

While over-expression of hQSOX1S reduced cell growth by about two-fold compared to control, knock down of QSOX1 increased cell growth by about two- fold.

The enhancement of clonogenic capacities is a specific characteristic to cancer cells. In order to evaluate the survival, proliferation capacities and self-renewal of our cell models, we performed clonogenicity tests [24]. After 21 days of standard culture conditions, QSOX1 overexpression led to 3.2-fold and 3-fold decreases of MCF-7 QSOX1S-1 and MCF-7 QSOX1S-2 colonies, respectively, compared to MCF-7 C (Figure 4C). In contrast, QSOX1 down expression enhanced the number of new colonies by 1.7- and 2-fold compared to MDA-MB-231 control cells (Figure 4D).

Inhibition of colony formation was correlated with the overexpression of QSOX1S while an enhancement of newly-formed clones was associated with QSOX1 knock down.

Another mechanism of tumor aggressiveness is the anchorage independent cell growth [25]. As such, we studied the role of QSOX1 on anchorage-independent growth. MCF-7 or MDA-MB-231 cell lines were plated in soft agar and incubated for two weeks before determining the size and number of colonies. The number of colonies decreased by 62.1% ± 7.2 (means ± SD) for QSOX1-1 and 64.4% ± 10.3 for QSOX1S-2 compared to the control (Figure 4E). Inversely, there was an enhancement of 158% ± 14.5 and 270% ± 8.5 colony number compared to shC for shQSOX1-1 and shQSOX1-2, respectively (Figure 4F). QSOX1 seems to play a role in the negative control of cell growth, anchorage independent cell proliferation and in the capacity to form new colonies.

### QSOX1 increased cell/matrix adhesion

**Figure 5:**
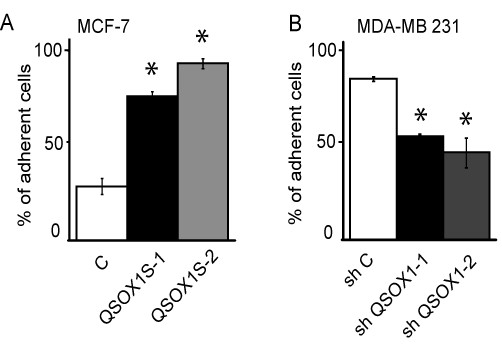
**Effect of QSOX1 cell/matrix adhesion**. MCF-7 (**A**) or MDA-MB-231 (**B**) cells were incubated at 37°C for 60 minutes at a density of 4 × 10^5 ^cells/well in serum-free DMEM. One hour later, adherent cell numbers were determined by counting Trypan blue excluding cells. Results were expressed as the ratio between cells adhered on the plate and total seeded cells and reported as the mean ± S.D. of triplicate determinations. * *P *<0.05, compared to control (Wilcoxon test). This experiment is representative of three separate assays.

Loss of cell adhesion to the matrix is often associated with tumor aggressiveness. We then tested cell adhesion to extracellular matrix by determining the number of adherent cells and cells remaining in the supernatant one hour after seeding. Figure 5 indicates the ratio between adherent cells and total seeded cells. Overexpression of QSOX1-S in MCF-7 cells led to a higher number of adherent cells, compared to MCF-7 C, one hour after seeding: 76% ± 2 (means ± SD), 93% ± 3 and 28% ± 4 of adherent cells in MCF-7 hQSOX1S-1, MCF-7 hQSOX1-2 and MCF-7 control, respectively (Figure 5A). Conversely, numbers of MDA-MB-231 shQSOX1-1 and shQSOX1-2 adherent cells were lower, compared to control: 52.4% ± 1, 44% ± 7 and 81.5% ± 1 of adherent, MDA-MB-231 shQSOX1-1, shQSOX1-2 cells, and shC respectively (Figure 5B). Interestingly, 24 h after seeding, no difference between the numbers of adherent cells in overexpressing or knock down models compared to their respective controls was observed (data not shown). These results suggested that QSOX1 could increase the speed of adhesion of cells to the extracellular matrix.

### QSOX1 decreased invasion

**Figure 6:**
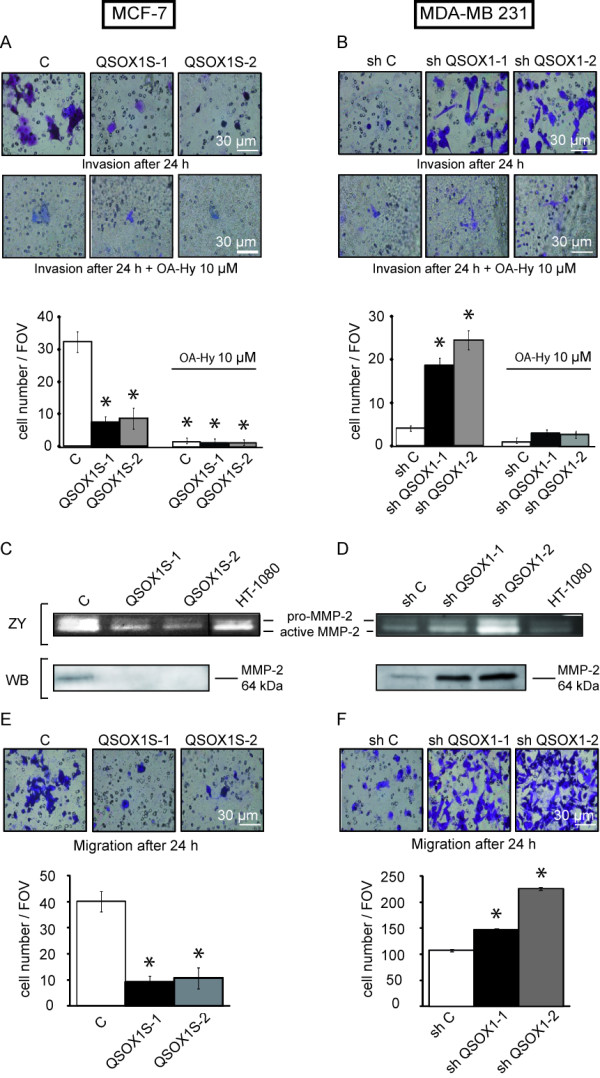
**Effect of QSOX1 on invasiveness**. (**A, B**) Cells were seeded on polycarbonate filter coated with Matrigel, in the presence/or not of a specific inhibitor of MMP-2 (OA-Hy) at 10 μM and incubated for 24 h and analyzed as described in Materials and Methods. Inserts were stained with 2% crystal violet and photographed. * *P *<0.05, compared to control (Wilcoxon test). (**C, D**)_ZY _Gelatin Zymography of matrix-metalloproteinase in conditioned medium. Cells were seeded and the media were collected after 24 h and 12 h of serum starvation for the MCF-7 and MDA-MB-231 cell lines respectively. Conditioned medium from HT-1080 cell line was used as positive control. Vertical line has been inserted to indicate a repositioned gel lane. (C, D)_WB _Blotting identification of MMP-2 in protein extracts from conditioned culture medium. (E, F) Cells were seeded on polycarbonate filters coated without ECM and incubated for 24 h and analyzed as described in (A). * *P *<0.05, compared to control (Wilcoxon test). These experiments are representative of three separate assays.

Since invasion is one of the key steps in tumor and metastasis development [26], it was studied with the modified "Boyden chamber" technique in the presence of Matrigel [27]. One day after seeding, 32 ± 3.1 (means ± SD) MCF-7 C cells were detected on the membrane's inferior face whereas only 7 ± 2 MCF-7 hQSOX1S-1 cells and 9 ± 3 MCF-7 hQSOX1S-2 cells were counted (Figure 6A). As regards the MDA-MB-231 model, only 4 ± 0.6 shC cells invaded the lower chamber compared to 19 ± 3 shQSOX1-1 cells and 25 cells ± 3 shQSOX1-2 cells through the membrane (Figure 6B). Thus, overexpression of QSOX1 in MCF-7 cells decreased cell invasion, whereas QSOX1 knock-down in MDA-MB-231 increased this cellular process. We also observed that invading cells with high QSOX1 expression were rounded, whereas those with low QSOX1 expression presented a stellate morphology (Figure 6A, B).

As degradation of the extracellular matrix by matrix metallo proteinases (MMPs) is characteristic of the invasion process [28], we studied the activity and secretion of MMP-2 and MMP-9, enzymes involved in the mechanism of invasion [29], by a zymography test and western blotting. Results in Figure 6C show the presence of pro and active MMP-2 in the MCF-7 C culture medium and an important decrease of intensity of the two bands in the MCF-7 QSOX1S-1 and MCF-7 QSOX1S-2 culture media (Figure 6CZ_Y_). Conversely, the bands corresponding to the pro and active MMP-2 were more intense in MDA-MB-231 shQSOX1-1 and shQSOX1-2 than in the control (Figure 6DZ_Y_). These results suggest that QSOX1 decreased both pro and active MMP-2 level in extracellular medium and thus the rate of MMP-2 activity. It is noteworthy to mention that MMP-9 activity was less intense in our models but the same activity variations as for MMP-2 were observed (data not shown). To determine if MMP-2 activity variations were due to a modulation of MMP-2 activation or to an increase in their expression or secretion, we analyzed the presence of this protein in the different culture media. As shown in Western blotting, Figure 6C, MMP-2 protein was detected in MCF-7 C conditioned medium extract but not in that of MCF-7 QSOX1S-1 and QSOX1S-2. Moreover, a very low level of MMP-2 was detected in MDA-MB-231 shC culture medium (Figure 6DW_B_) whereas it was greatly increased in those of MDA-MB-231 shQSOX1-1 and MDA-MB-231 shQSOX1-2. Quantification of MMP-2 mRNA by RTqPCR was performed but neither the overexpression or knock-down of QSOX1 influenced MMP-2 mRNA level (data not shown). Thus, QSOX1 could attenuate MMP-2 extracellular activity by probably acting on its stability and/or its secretion.

In order to examine if the observed differences in invasion assays were associated with a modulated activity of MMP-2, we performed invasion assays in the presence of a specific inhibitor of MMP-2. One day after seeding, 1.5 ± 0.6 (means ± SD) MCF-7 C cells were detected on the membrane's inferior face and, as MCF-7 C, only 1.25 ± 0.5 MCF-7 hQSOX1S-1 cells and 1.25 ± 0.5 MCF-7 hQSOX1S-2 cells were counted (Figure 6A). With respect to the knock-down model, 1.3 ± 0.6 shC cells invaded the lower chamber and 3.7 ± 0.6 shQSOX1-1 cells and 3.3 cells ± 0.6 shQSOX1-2 cells passed through the membrane (Figure 6B). Inhibition of MMP-2 results in a loss of all differences in variations of invasion between different cancer cell models. These results show that QSOX1 down-regulates invasion, probably in part by negatively regulating MMP-2 activity.

Since the capacity of cells to migrate through a physiological membrane is also a component of invasion, cell migration was studied using the modified "Boyden Chamber". Cells were seeded in a Boyden chamber lacking the Matrigel layer and cells able to cross the membrane were counted 24 h later. Cell number was 40 ± 6 (means ± SD) for MCF-7 C, and 9 ± 3 and 11 ± 4 for MCF-7 hQSOX1S-1 and MCF-7 hQSOX1S-2, respectively (Figure 6E). In our knock-down model, there were only 108 ± 2 shC cells that passed through the membrane, whereas 147 ± 1 shQSOX1-1 cells and 226 ± 3 shQSOX1-2 cells were counted. QSOX1 knock-down in MDA-MB-231 cells increases the cellular motility (Figure 6F). Interestingly, the extent of the phenotypes observed in our knock-down models was inversely related to QSOX1 expression levels. Thus, it appears that QSOX1 also reduced cell migration.

### QSOX1 suppressed tumor development *in vivo*

**Figure 7:**
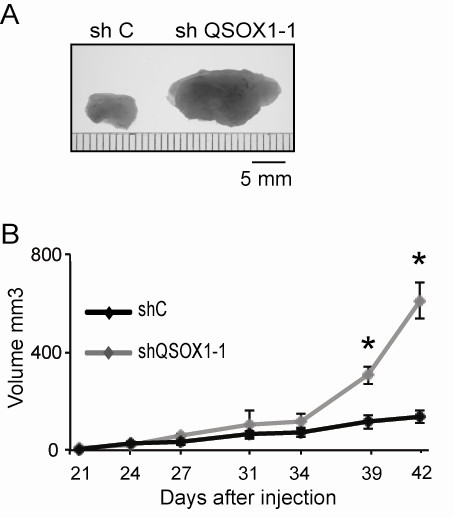
**QSOX1 reduces breast cancer xenograft formation**. 2.10^6 ^MDA-MB-231 shC and MDA-MB-231 shQSOX1-1 cells were injected subcutaneously in CIEA NOG mice (*n *= 5 per group). Tumor volume was calculated by the formula *V = *½ *a × b^2^*, where *a *is the longest tumor axis, and *b *is the shortest tumor axis. (**A**) 42 days after injection, tumors were fixed in formol and photographed, (**B**) 21 days after injection, the evolution of the tumor volume was measured biweekly. * *P *<0.05, compared to control (Wilcoxon test). This experiment is representative of two separate assays.

Tumorigenic properties of the QSOX1 knock-down models were analyzed in CIEA NOG mice. Tumor size was monitored for two months after inoculation. Results showed that decreased expression of QSOX1 led to a strong enhancement of the size (Figure 7A) and growth of the tumors (Figure 7B). In fact, 42 days after inoculation, the mean volume of MDA-MB-231 shQSOX1-1 tumors was 2.65 times higher than the mean volume of tumors obtained in the CIEA NOG control mice. Thus, *in vivo*, QSOX1 drastically reduced tumor growth. In addition, during the excision of tumors produced from the cell line MDA-MB-231 shC, we observed that tumors had developed just under the skin of animals while tumors from the MDA-MB-231 shQSOX1-1 cell line had developed in the subjacent muscle tissue (Additional file 1). This suggests that QSOX1 could be unfavorable for tumor invasion.

## Discussion

In this study, we provide, for the first time, an insight into the *QSOX1 *expression level in breast tumors and into its role in breast cancer cells and tumor development.

Even if the enzymatic function of QSOX1 is well described in the literature, its biological function is not clearly established. In fact, QSOX1 expression is differentially modulated in some cancers. It turns out that QSOX1 is overexpressed during the early stages of prostate cancer, and in pancreatic tumor cells [15-17]. However, it has also been shown that QSOX1 is down-regulated in an endothelial cancer cell model [18]. As such, we decided to investigate the QSOX1 expression in normal and cancerous human breast tissues.

Prior to our study, it was not previously reported where QSOX1 was expressed in breast tissues. IHC results showed that the protein was expressed in the endomembranous system in epithelial cells, but not in stroma cells and in adipocytes. Results were consistent with the fact that QSOX1 is a protein specifically expressed in epithelial cells and secreted [1,30]. The differential localization of QSOX1 in normal and cancer cells could be due to post-translational modifications and/or a differential regulation of the expression of QSOX1 isoforms. The signal surrounding tumor cells could also be due to an epithelial cell depolarization, causing a loss of punctuate labeling and the appearance of a diffuse staining. Moreover, QSOX1 is expressed in pancreatic tumor cells and not in non-cancerous peripheral cells [16]. Together, these results indicate that QSOX1 is not constitutively expressed in different tissues, suggesting that the cellular role for this protein may also be tissue-dependent.

As QSOX1 was expressed in breast cancer tissues, we assessed if its expression level could be correlated to the outcome of breast cancer patients. Thus, we studied the QSOX1 mRNA expression in a retrospective cohort of 217 invasive ductal carcinomas of the breast. It appears that breast tumors presenting good prognostic criteria, such as low histological grade and steroid receptor-positive status, express a high level of QSOX1 mRNA (Figure 1A and Table 1). Furthermore, Kaplan-Meier curves (Figure 1B) showed that patients with high QSOX1 levels presented a significantly better metastasis-free survival compared to patients with low QSOX1 mRNA levels. These data suggest that the QSOX1 expression level could negatively correlate with the aggressiveness of breast tumors.

To determine the extent of association between QSOX1 expression and breast cancer patient outcome, we investigated the roles of QSOX1 on characteristic cancer cell phenotypes in MCF-7 overexpressing QSOX1 and MDA-MB-231, in which QSOX1 expression is knocked down. It must be underlined that QSOX1 levels are higher in the invasive MDA-MB-231 cells compared to the more indolent MCF7 cells. Differences between these two cell lines are not limited to basal QSOX1 expression but also include the expression of several genes, such as steroid hormone receptors and the receptor 2 of the epidermal growth factor [31]. Therefore, QSOX1 expression could not be responsible in itself for the difference in aggressiveness of these cell lines.

After validation of our cellular models, we investigated the role of QSOX1 on proliferation, clonogenic capacities and invasion. Above all, it is interesting to note that all observed phenotypes obtained with the overexpression of QSOX1 were opposite of those obtained with the QSOX1 knocked-down model. Moreover, in the latter cell model, the intensity of the effect was correlated to the extent of QSOX1 knock-down.

We report in this study that QSOX1 inhibits breast cancer cell proliferation. These data are in accordance with previous studies showing that QSOX1 decreased epithelial and endothelial cell proliferation [11,18]. The fact that QSOX1 was induced in quiescent WI38 fibroblasts, but not in cycling lung fibroblasts, suggests that it could act in the negative control of the cell cycle and reinforces the idea that it could downregulate cell proliferation [3]. Conversely, Katchman and coworkers suggested that a QSOX1 knock-down in different pancreatic cancer cells decreased proliferation. But, cell cycle analyses did not give clear conclusions about QSOX1 role on cell cycle control [16]. All these results suggested that QSOX1 role on proliferation and cell cycle could be cell type or tumor stage dependent.

The study of cancer cell proliferation is generally performed in parallel with the study of the cell clonogenic capacity, often associated with tumor development and establishment of metastases [32]. Thus, the fact that QSOX1 reduced cell clonogenicity and anchorage-independent growth is in accordance with our observations on proliferation. In addition, we studied the effect of QSOX1 on cell-matrix adhesion, which is often disrupted in breast cancer. Our findings demonstrate that QSOX1 increased cell adhesion to the extracellular matrix but more specifically early in the adhesion process. Since QSOX1 is implicated in protein folding, we can hypothesize that QSOX1 could enhance the folding or the addressing of newly synthesized proteins implicated in cell adhesion to the matrix. In fact, it is already known that integrin activation can be mediated by thiol disulfide exchanges within the extracellular domain of the beta subunit [33,34], a process often performed by the Protein Disulfide Isomerase (PDI) [35,36]. QSOX1 could activate integrins directly by creating a thiol-disulfide bond or indirectly via an oxidation of PDI [9].

We showed in our retrospective cohort of invasive ductal carcinomas of the breast that a high QSOX1 expression is associated with low expression of a well-established marker of migration and invasion phenotype in breast cancer: PAI1 (Table 1) [37]. In fact, the tumor cell must first degrade basement membrane components before being able to migrate and establish itself in another organ [38]. We demonstrated that QSOX1 decreased the ability of cells to invade the Matrigel. Furthermore, a low QSOX1 expression level confers to the invading cells this stellate morphology described as characteristic of a high metastatic potential [39].

Considering that QSOX1 decreased cellular invasion, we studied the activity of MMP-2 and MMP-9, enzymes widely described to be involved in the mechanism of invasion [29]. QSOX1 decreased both pro and active MMP-2 level in extracellular medium and thus the rate of MMP-2 activity. QSOX1 has also a lesser effect on MMP-9 activity. Nevertheless, QSOX1 had no effect on other MMPs implicated in the basal membrane degradation process, such as MMP-3 and MMP-7 [29] (data not shown). It seems that QSOX1 reduced invasion by down-regulating MMP-2 activity, since the effect of QSOX1 on invasion was no longer observable in the presence of a specific MMP-2 inhibitor. The involvement of MMP-2 in cell proliferation [40] could account for our results on this cellular process.

The MMP-2 regulation is very complex and the role of QSOX1 on this MMP remains to be determined. Our results suggest that QSOX1 could attenuate MMP-2 extracellular activity by probably acting on MMP-2 secretion and/or stability without modifying the mRNA level.

Thus, QSOX1 could inhibit MMP-2 activation by promoting cell matrix adhesion. In fact, when cells do not adhere to the matrix, integrin αvβ3 interacts with MMP-2 and so participates to its activation [29]. The TIMPs (Tissue Inhibitors of MetalloProteinases), present six conserved disulfide bonds involved in their interaction domains with MMPs [41]. QSOX1 could promote disulfide bond formation in TIMPs, and thus their interaction with MMP-2, leading to its inhibition.

Interestingly, the activation and secretion of MMP-2 could also be positively regulated by oxidative stress through the oxidation of its pro-peptide [41]. Since QSOX1 protected cell against oxidative stress [11], it could eventually prevent oxidation of the pro-peptide and so MMP-2 secretion and activation.

Conversely, it has been shown in pancreatic cancer cells that QSOX1 favored the activities but not the secretion of MMP-2 and MMP-9; whereas, QSOX1, in our breast cancer cell models, decreases both the activity and secretion of these MMPs. The discrepancies between our results on breast cancer cells and Katchman's results on pancreatic cells could be explained by differential expression of QSOX1 isoforms and its substrates availability. Furthermore, QSOX1 participates to the redox state control together with a network of proteins like PDI, Ero1, Gluthatione reductase or thioredoxins. It would be interesting to know the expression level of all these actors to understand how QSOX1 could generate opposite effects.

Given these conflicting results between the role of QSOX1 in the breast and pancreas cancer cell models, we performed an *in vivo *study. We demonstrated that QSOX1 drastically inhibited tumor growth. Moreover, it should be noted that during the tumor resection, we observed that QSOX1 was unfavorable for tumor invasion in subjacent muscle tissue. By inhibiting MMP-2, an enzyme involved in tumor development, angiogenesis and metastasis [42], QSOX1 could disfavor breast tumor development and aggressiveness. Indeed, it has been demonstrated in endothelial cancer cell models that QSOX1 could inhibit angiogenesis [18]. It would consequently also be interesting to study angiogenesis to better understand the effect of QSOX1 on tumor growth through MMP-2 activity regulation.

## Conclusion

In our study, we demonstrate that QSOX1 could inhibit breast tumor development and aggressiveness, which is in agreement with our findings, indicating that a high QSOX1 expression is associated with a better survival for breast cancer patients. QSOX1 could be regarded as a new marker of good prognosis. All our data provide new insights into the role of QSOX1 in cancer cell biology.

## Abbreviations

DMEM: Dulbecco's Minimum Essential Medium; ECM: Extra Cellular Matrix; ER: Estrogen Receptor; ERV/ALR: Essential for Respiration and Vegetative growth/Augmenter of Liver Regeneration; FAD: Flavin Adenine Dinucleotide; FCS: Fetal Calf Serum; GAPDH: GlycerAldehyde-3-Phosphate DeHydrogenase; HRP: HorseRadish Peroxidase; HUVEC: Human Umbilical Vein Endothelial Cell; IDC: Invasive Ductal Carcinomas; MCF-7: Michigan Cancer Foundation-7; MDA-MB-231: M.D Anderson-Metastatic Breast-231; MDA-MB-453: M.D Anderson-Metastatic Breast-453; MMP-2: Matrix MetalloProteinase-2; OA-Hy: *cis-*9-OctAdecenoyl-N-Hydroxylamide; PAI-1: Plasminogen Activator Inhibitor-1; PDI: Protein Disulfide Isomerase; PgR: Progesterone Receptor; PVDF: PolyVinyllidene DiFluoride; QSOX1: Quiescin/Sulfhydryl OXidase 1; ROS: Reactive Oxygen Species; RTqPCR: Reverse Transcription quantitative Polymerisation Chain Reaction; SBR: Scarff, Bloom and Richardson; SDS: Sodium DodecylSulfate; SDS PAGE: SDS PolyAcrylamide Gel Electrophoresis; shRNA: Small Hairpin RNA; TIMP: Tissue Inhibitor of MetalloProteinases; TBST: Tris Buffered Saline Tween

## Competing interests

The authors declare that they have no competing interests.

## Authors' contributions

NP performed research, analyzed data and wrote the paper. FH performed research and analyzed data. PA participated in data interpretation and in the writing of the paper. AV participated in experiment design. FD performed clinical data analysis and interpretation. MA performed clinical RT-qPCR analysis. CB provided his expertise in *in vivo *cancer experiments. JRP performed *in vivo *experiments execution. GV performed immunohistochemistry data analysis and interpretation. FE provided critical reagents. MJ participated in writing of the manuscript. GD designed research, analyzed data and wrote the paper. All authors have read and approved the manuscript for publication.

## Supplementary Material

Additional file 1 **Tumor localization during their excision. **During the excision of tumors (white arrow), tumors from the cell line MDA-MB-231 shC developed just under the skin of animals while tumors from MDA-MB-231 shQSOX1-1 developed in the subjacent muscle tissue. Click here for file
