# Supplementary figures and images for: High expression of QSOX1 reduces tumorogenesis, and is associated with a better outcome for breast cancer patients
Source: Breast Cancer Res. 2012 Oct 25;14(5):R136. doi: 10.1186/bcr3341 (PMC4053115; doi:10.1186/bcr3341)

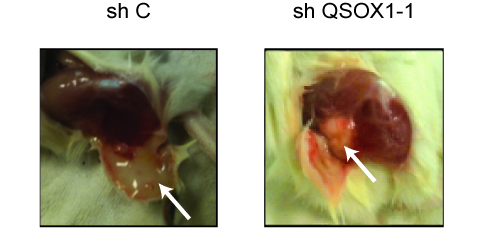

Supplement: Additional file 1 — Tumor localization during their excision. During the excision of tumors (white arrow), tumors from the cell line MDA-MB-231 shC developed just under the skin of animals while tumors from MDA-MB-231 shQSOX1-1 developed in the subjacent muscle tissue. [file bcr3341-S1.TIFF]
